# Antimicrobial potential of four mica drugs and their chemical and mineralogical properties

**DOI:** 10.1186/s12906-022-03545-w

**Published:** 2022-03-11

**Authors:** Apsara Wijenayake, Charmalie Abayasekara, Amarasooriya Pitawala, B. M. Ratnayake Bandara

**Affiliations:** 1 Postgraduate Institute of Science, University of Peradeniya, Peradeniya, Sri Lanka; 2 Department of Geology, Faculty of Science, University of Peradeniya, Peradeniya, Sri Lanka; 3 Department of Botany, Faculty of Science, University of Peradeniya, Peradeniya, Sri Lanka; 4 Department of Chemistry, Faculty of Science, University of Peradeniya, Peradeniya, Sri Lanka

**Keywords:** Mica drugs, *Bhasma*, Traditional medicine, *Rasashastra*, Antimicrobial, Biotite

## Abstract

**Background:**

Mica drugs, a group of herbo-metallic traditional preparations comprising biotite mica as the major mineral ingredient, are prescribed for skin disorders and respiratory ailments and other chronic conditions in South Asian countries, particularly India and Sri Lanka. Mica-based drugs (*Abhrak* drugs) are subjected to unique and varied preparation procedures and the bioactivity of the drugs can be affected by drug-processing conditions, the ingredients used and the mica composition. The current study aimed to evaluate and compare, on the basis of their physical and chemical characteristics, the antimicrobial potential of two commercial mica drugs AbBb (*Abhrak bhashma*) and AbCh (*Abhrak Chenhuram*) and two mica drugs ABL1 (*Abhrak Bhasma* Laboratory Prepared 1) and ABL2 (*Abhrak Bhasma* Laboratory Prepared 2) prepared in the laboratory under different conditions.

**Methods:**

Antimicrobial activity of all four drugs was assessed at 10 mg/ml concentration against *Pseudomonas aeruginosa*, *Escherischia coli, Staphylococcus aureus*, methicillin-resistant *S. aureus* (MRSA) and *Candida albicans* using well diffusion assay, agar dilution assay and Miles and Misra method. Major and trace metal constituents of the drug samples were measured using atomic absorption spectrometry. Mineralogical properties, bacteria-mineral interactions, morphological changes in microbes and the surface characteristics of the drugs were determined using X-ray diffraction (XRD) analysis and scanning electron microscopy (SEM).

**Results:**

The drugs ABL1, ABL2 and AbBh exhibited antimicrobial activity against only Gram-positive organisms (*S. aureus* and MRSA) when tested with Miles and Misra method (broth method). Mineralogical studies (XRD) revealed that biotite mica was altered into secondary clay minerals and iron oxides in the commercial drug AbCh while the other three drugs had altered mica and iron oxide phases. The essential elements (Na, K, Ca and Mg) required for microbial functions were present in varying extents in all four drugs while they were present in exceedingly high amounts in AbCh having comparatively high cation-exchange capacity, consistent with the observation that AbCh was inactive against all the microbes tested. The three drugs (ABL1, ABL2 and AbBh) showing antimicrobial activity contained comparatively high amounts of Fe, Zn and Cu that are known to display antimicrobial properties at high concentrations. SEM studies revealed that the drug particles adhered and entrapped the bacterial species, presumably modifying the physiochemical characteristics of the bacteria and eventually causing lethality.

**Conclusion:**

Three of the four mica drugs inhibited the tested Gram-negative bacteria and the antibacterial activity of the mica drugs depends on their constituents and the methods of preparation.

## Introduction

The increasing antibiotic resistance exhibited by pathogenic microbes has prompted efforts to discover new antimicrobials covering a diverse range. Researchers have increasingly paid attention towards traditional medicine, to find out novel sources of antimicrobials [1, 2]. From ancient times, mankind has discovered the healing potential of certain minerals against microbes besides the use of herbs as medicine [3–5]. The use of minerals for healing purposes is widespread in Indian, Chinese and African traditional medicinal systems [6, 7].

‘Mica Ash’ is an example for a herbo-metallic traditional preparation comprising biotite mica (as the major mineral ingredient), plant materials and other ingredients. Mica ashes (drugs) are prescribed in traditional medicines in South Asian countries (specially in India and Sri Lanka) for skin disorders, respiratory and other chronic diseases [8, 9].

Although mica drugs are being constantly used in alternative medicinal applications, their importance for the development of allopathic drugs has not been sufficiently assessed. Some of the limitations such as inert nature of mica and poor bioavailability can hinder the use of mica in drugs. Further, there can be risks associated with prolonged oral administration of mica drugs due to the presence of toxic metal constituents in the raw materials [10, 11].

Nevertheless, utilization of ground mica or synthesized mica products in modern cosmetics [12, 13] implies their potential dermatological applications in treating diseases caused by microorganisms. The current study attempts to analyze the possible topical applications of selected mica-based traditional formulations, for the potential of these drugs to be developed as antimicrobial agents.

It is well known that the cationic constituents, surface nature and cationic exchange properties of minerals can influence their antimicrobial properties [14–16]. Evaluation of antimicrobial properties of mica ash products is important because mica ashes consist of nano- to micro-scale particles, which are enriched with cations possessing antimicrobial activity [17, 18].

However, antimicrobial properties of mica ash-based drugs can depend on the conditions involved in traditional drug-processing techniques [17, 19] the composition of mica and the other ingredients [4]. Moreover, these techniques and ingredients are not adequately specified for commercial products. The main objective of the current study was to evaluate antimicrobial activity of two commercial mica drugs and two drugs prepared in the laboratory under variable conditions, against Gram-positive and Gram-negative bacteria and a fungal species, and to seek correlations with constituent cations, surface morphology and cation-exchange capacity of the drugs.

## Materials and methods

### Drug samples

Two ‘*Rasashastra’* drugs, designated ABL1 (*Abhrak Bhasma* Laboratory Prepared 1) and ABL2 (*Abhrak Bhasma* Laboratory Prepared 2), were prepared in the laboratory, using biotite mica as the mineral ingredient. Biotite mica was subjected to specific alteration processes as in traditional preparations [20], which included heating, quenching and incineration with additional ingredients. Table 1 summarizes the conditions employed in the preparation of drugs. A muffle furnace was used for heating the samples and cow’s urine for quenching. The drugs were ground using a ball mill (Fritsch Pulverisette) and particles less than 125 μm were used in the current study.

**Table 1:** Raw ingredients and preparation conditions of the laboratory-prepared and commercial drugs^a^

Drug	Ingredients	Heating temperature/ cycles	Quenching medium/ cycles	Temperature/ incineration cycles
ABL1	Biotite mica, *Ficus benghalensis* leaves, *Ricinus communis* water extract and jaggery	1000 °C/7	Cow urine, vinegar 3 days/7	1000 °C/10
ABL2	Biotite mica, sulfur	1000 °C/7	Cow urine, vinegar 3 days/7	1000 °C/1
AbBh	Biotite mica + unknown	unknown	unknown	1000 ^°^C/1
AbCh	Biotite mica + unknown	unknown	unknown	1000 °C/30

^a^ABL1 and ABL2 were formulated in the laboratory while AbBh and AbCh were commercial products

Two traditional mica drugs, namely *Abhrak Bhasma* (AbBh, a *Rasashastra* drug) and *Abhrak Chenhuram* (AbCh*,* a *Siddha* drug), were purchased from Dabur India Ltd. and Indian Medical Practitioners’ Co-operative Pharmacy and Stores Ltd., respectively. According to traditional medical records, the method of preparation for AbBh is different from that for AbCh although biotite mica was the mineral ingredient in both drugs [8, 21]. The organic and other inorganic ingredients used in the commercial drug preparations are unknown (Table 1).

### Antimicrobial assays

#### Microbial strains

The microorganisms assessed included two Gram-positive bacteria [*Staphylococcus aureus* (ATCC 25923) and methicillin-resistant *Staphylococcus aureus* (MRSA)], two Gram-negative bacteria [*Pseudomonas aeruginosa* (ATCC-27853) and *Escherichia coli* (ATCC 25922)] and one fungal species *Candida albicans* (ATCC 90028), which were obtained from the culture collection of the Department of Microbiology, Faculty of Medicine, University of Peradeniya, Sri Lanka.

#### Preliminary semi-quantitative assays (well diffusion and agar dilution assays)

All four drugs were assessed for antimicrobial activity using the well diffusion assay and agar dilution assay. Fine powders of the drugs were suspended separately in diluted dimethyl sulfoxide (1:7, DMSO:water). Each drug and its components were tested (at 10 mg/ml concentration) against the microbes mentioned above. Inoculum of each microbial strain was prepared by suspending the respective 18- to 24-h old culture in aqueous NaCl (0.85%). Turbidity of each inoculum was adjusted to 0.5 McFarland standard.

Well diffusion assay for each microbial strain was done by flooding the inoculum onto the surface of a Petri plate containing 25 ml of Mueller-Hinton agar (MHA). Wells were bored into agar using a sterile cork borer with a diameter of 9 mm. The bases of the wells were sealed by adding a drop of molten agar into each well. Aliquots (180 μl) of the drug suspensions (10 mg/ml) were added into separate wells. After incubation overnight at 37 °C, the plates were examined for zones of inhibition. Diluted DMSO was used as the negative control.

Agar dilution plates were prepared by mixing sterilized and cooled molten agar with each drug separately. Final concentration of each drug was 10 mg/ml in each test plate. Agar plates containing the drugs were kept until solidified and 5 μl suspension from each organism was placed on the dried agar surface of each plate. After incubation overnight at 37 °C, the plates were examined for microbial growth on the drug bearing agar medium.

#### Quantitative antimicrobial assays (miles and Misra method)

Inoculated Brain Heart Infusion (BHI) broth cultures of turbidity similar to 0.5 McFarland standards of the above microorganisms were mixed with each drug (10,000 ppm = 10 mg/ml) and incubated at 37 °C for 18–24 h. The experimental vials were kept in a horizontal shaker during the time of incubation for thorough mixing of the drugs and to avoid deposition of drugs at the bottom of the growth medium. The antimicrobial activity of each drug was quantitatively investigated using Miles and Misra method for viability [22] after incubation as follows: a 100-μl aliquot from each of the experimental vials was serially diluted and 20 μl from each dilution was placed on MHA medium, which was designated into 6 sectors. The plates were incubated at 37 °C for 24 h. After incubation, colony forming units (cfu) were recorded in the respective dilutions of all the drugs.

The above experimental vials containing the broth and drugs were centrifuged for 1 min at 300 rpm [23] to enable drug particles to settle at the bottom of the vial; any settling of some of the microbes during centrifugation was common and considered almost same in all the vials including controls. The supernatants were used to determine the turbidity by means of absorbance at 625 nm, using UV-visible spectrophotometer (SHIMADZU-UV 1800). The antimicrobial experiments were replicated 3 times with an initial concentration of 10 mg/ml of each drug. Absorbance of the drug controls and the organism controls (organism without the drug) was measured to deduce the effective turbidity generated in the treated vials.

#### Mineralogical and chemical characterization

Mineralogical characterization of the drug samples was performed using Siemens D-5000 X-ray diffraction (XRD) instrument which was operated using CuKα radiation at 40 kV and 34 mA. Each drug sample was digested with hydrochloric, nitric, perchloric and hydrofluoric acids as described by Gaudino et al. [24, 25] for chemical analysis. Selected major and trace metal cation concentrations were measured on Perkin Elmer-2800 Atomic Absorption Spectrophotometer (AAS). The analyses were done in triplicates together with the reagent blanks and averaged values were used to compare the drug samples. Detection limits of AAS for the measured elements varied 0.01–0.004 ppm.

#### Cation-exchange Capacity (CEC)

The CEC was determined by mixing the drug samples (1.000 g) with an excess of 1 M sodium acetate solution (100 ml) to exchange with the mobile interlayer cations in the mica drugs. Na^+^-saturated samples were then washed three times with isopropyl alcohol to remove excess sodium ions. 1 M ammonium acetate solution (100 ml) was added to replace the adsorbed sodium ions with ammonium ions. The removal of sodium ions was assessed by AAS analysis [26].

#### Scanning Electron Microscopy (SEM) analysis

SEM analysis (SEM-EVO-LS15) of drug samples and bacteria was carried out separately and in combination to determine the bacteria-mineral interactions, morphological changes in microbes and the surface characteristics of the drugs. Prior to loading the samples for imaging, all the treated samples and untreated bacterial samples (blank) were fixed with formaldehyde at room temperature for 2 h, washed three times with sterilized water and dehydrated twice with an ethanol series (30, 50, 70, 80, 90 and 100%). The samples were kept at each ethanol solution for 10 min [27, 28]. To minimize artifacts during imaging, all samples were observed under variable pressure mode.

#### Assessment of cytotoxicity

To investigate the cytotoxicity of the four drugs, brine shrimp lethality bioassay [29–32] was carried out as follows: brine shrimps (*Artemia salina*) eggs were hatched in artificial seawater under continuous aeration for 48 h. After hatching, 10 nauplii were drawn through a glass capillary and placed in each vial containing 5 ml of brine solution to which the drug preparation was added. Each experiment was conducted along with the untreated shrimp control. All the tested brine solutions were maintained at room temperature under mild shaking conditions. Survival of brine shrimp nauplii at each concentration was recorded after 12, 18 and 24 h; experiments were conducted for different concentrations of the drugs ranging 1–10 mg/ml. All experiments were triplicated.

## Results

### Antimicrobial assays on the drugs

**Fig. 1:**
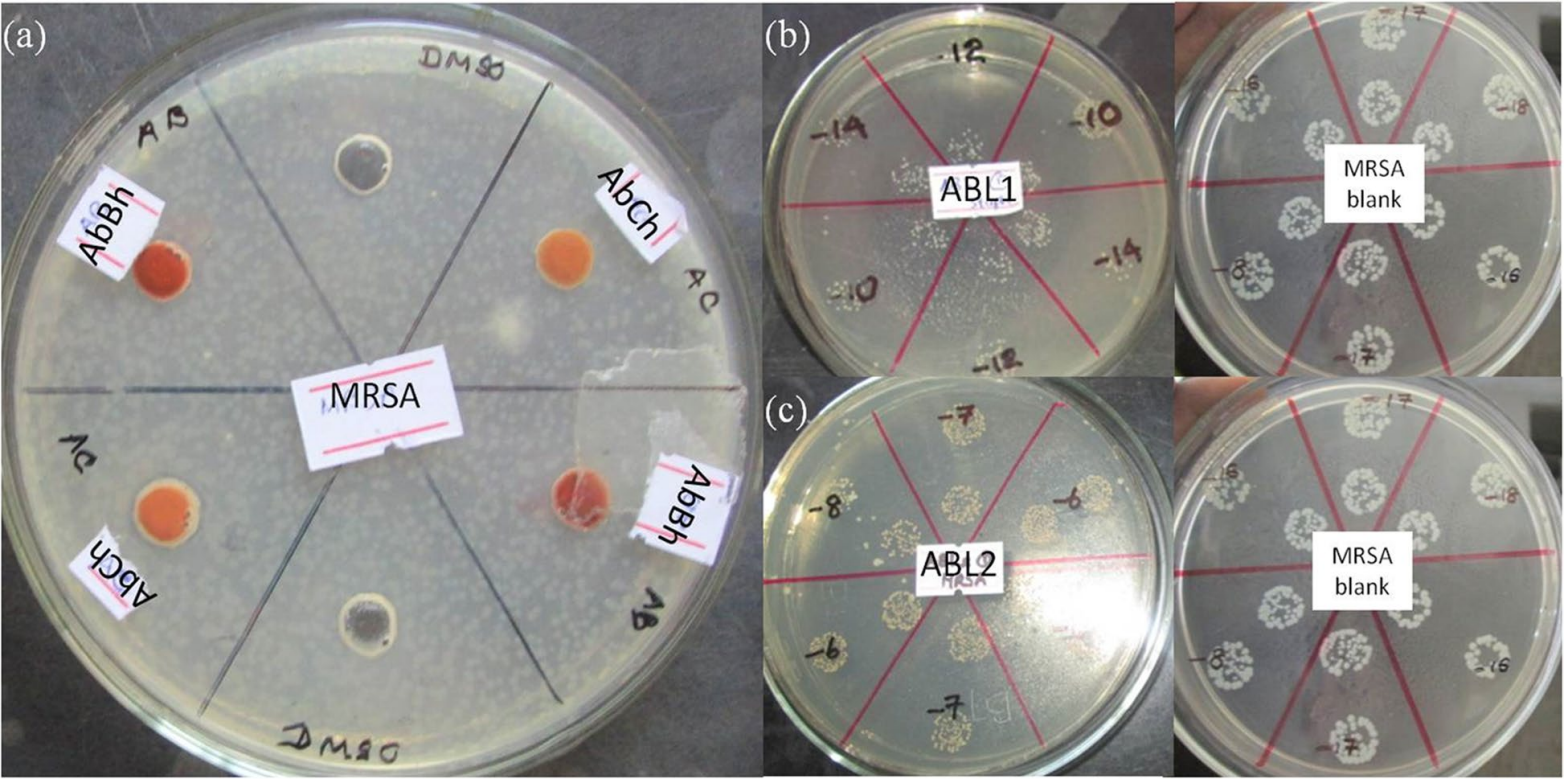
(**a**) Well diffusion assay for AbBh and AbCh drugs against MRSA (**b**) antimicrobial effect of ABL1 and (**c**) ABL2 drug as determined by Miles and Misra method at 10 mg/ml concentration against MRSA. Note the reduction in cfu compared to the organism control (extreme right)

All four drugs did not show activity in the agar well diffusion assay (Fig. 1a) nor in the agar dilution assay. Inhibitory activity was observed with ABL1, ABL2 (Fig. 1b and c) and to a lesser extent with AbBh against *S. aureus* and MRSA in the Miles and Misra method (Table 2). AbCh did not exhibit antimicrobial activity. Although ABL1 and ABL2 were prepared from the same mica source, antimicrobial activity of the drugs showed different activity against *S. aureus* and MRSA, indicating that the ingredients used and the method of preparation influence the antimicrobial effect of the drug. None of the drugs showed antibacterial activity against *P. aeruginosa*, *E. coli* and *C. albicans.*

**Table 2:** Viable cell count (cfu/ml) by Miles and Misra method and respective absorbance values of broth cultures that contained ABL1, ABL2, AbBh and AbCh drugs at 10 mg/ml concentration

Microganism	Viable cells (CFU/ml)					Absorbance				
	ABL1	ABL2	AbBh	AbCh	Control	ABL1	ABL2	AbBh	AbCh	Control
*P. aeruginosa*	10^15^	10^15^	10^15^	10^15^	8 × 10^15^	1.71	1.75	1.69	1.65	1.65
*E. coli*	10^18^	10^18^	10^18^	10^18^	8 × 10^18^	1.84	1.87	1.86	1.88	1.86
*S. aureus*	^a^8x10^7^	17 × 10^8^	29 × 10^9^	20 × 10^15^	2 × 10^15^	^a^0.6	0.6	0.6	1.61	1.6
MRSA	15 × 10^11^	14 × 10^12^	4 × 10^9^	37 × 10^17^	9 × 10^17^	1.6	1.7	1.5	1.90	1.8
*C. albicans*	21 × 10^6^	43 × 10^6^	17 × 10^6^	52 × 10^6^	4 × 10^6^	0.99	0.96	1.0	0.97	0.90

^a^Boxed values indicate antimicrobial activity of the respective drugs against a particular organisam

Absorbance measurements of the test organisms reflecting microbial density (Miles and Misra method) are given in Table 2. Reductions in absorbance were shown for *S. aureus* and MRSA by ABL1, ABL2 and AbBh indicating growth inhibition of bacteria. AbCh showed an increment in absorbance compared with the organism control for all the microbial species tested, indicating a growth enhancement rather than an inhibition.

### Characterization of drug samples

#### Mineralogical characterization

**Fig. 2:**
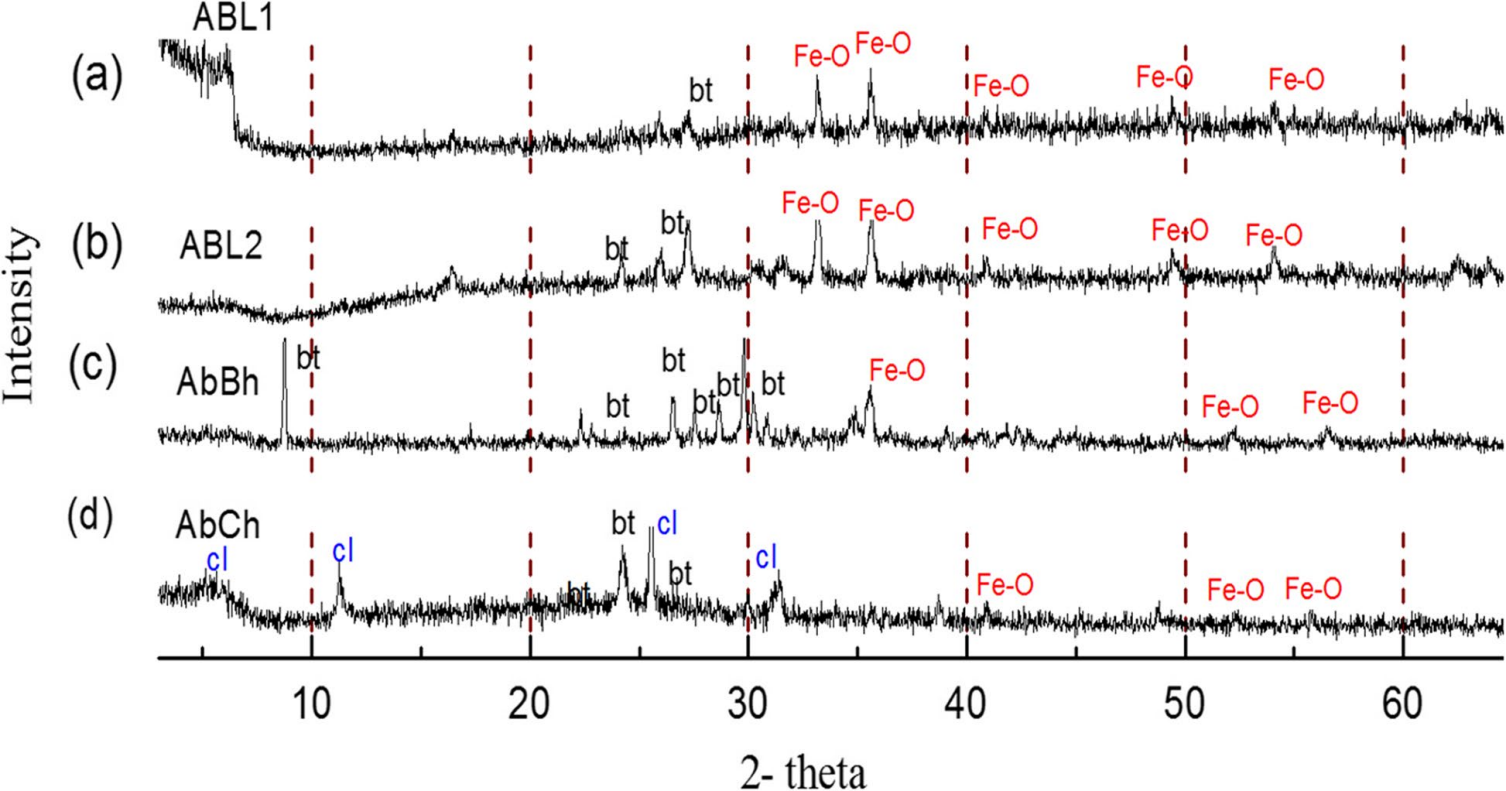
X-ray diffraction patterns of (**a**) ABL1, **b** ABL2, **c** AbBh and **d** AbCh drugs. bt – biotite; (Fe-O) -iron oxide; cl – clay mineral

XRD patterns (Fig. 2a-d) of drug samples showed that all the samples consisted of unaltered biotite mica and iron oxide as main crystalline phases. For each drug, the peak intensities of the phases varied reflecting the amounts of corresponding phases in a particular drug. Although biotite was a major ingredient in the preparation of all four drugs, its presence was clearly detected only in AbBh (Fig. 2c), which had been subjected to minimal thermal alteration (a single incineration cycle only). Other drug samples (ABL1, ABL2 and AbCh) showed minor amounts of unaltered biotite reflecting the modification of biotite during drug preparation. XRD patterns also indicate that the number of cycles of heating and incineration had influenced the formation of amorphous materials and some amounts of iron oxide phases. Further, the XRD data indicate that the mineral phases of ABL1 and AbCh (Fig. 2a and d) had been significantly altered and secondary clay minerals had been formed in AbCh (Fig. 2d).

#### Chemical constituents and Cation-Exchange Capacity (CEC)

**Fig. 3:**
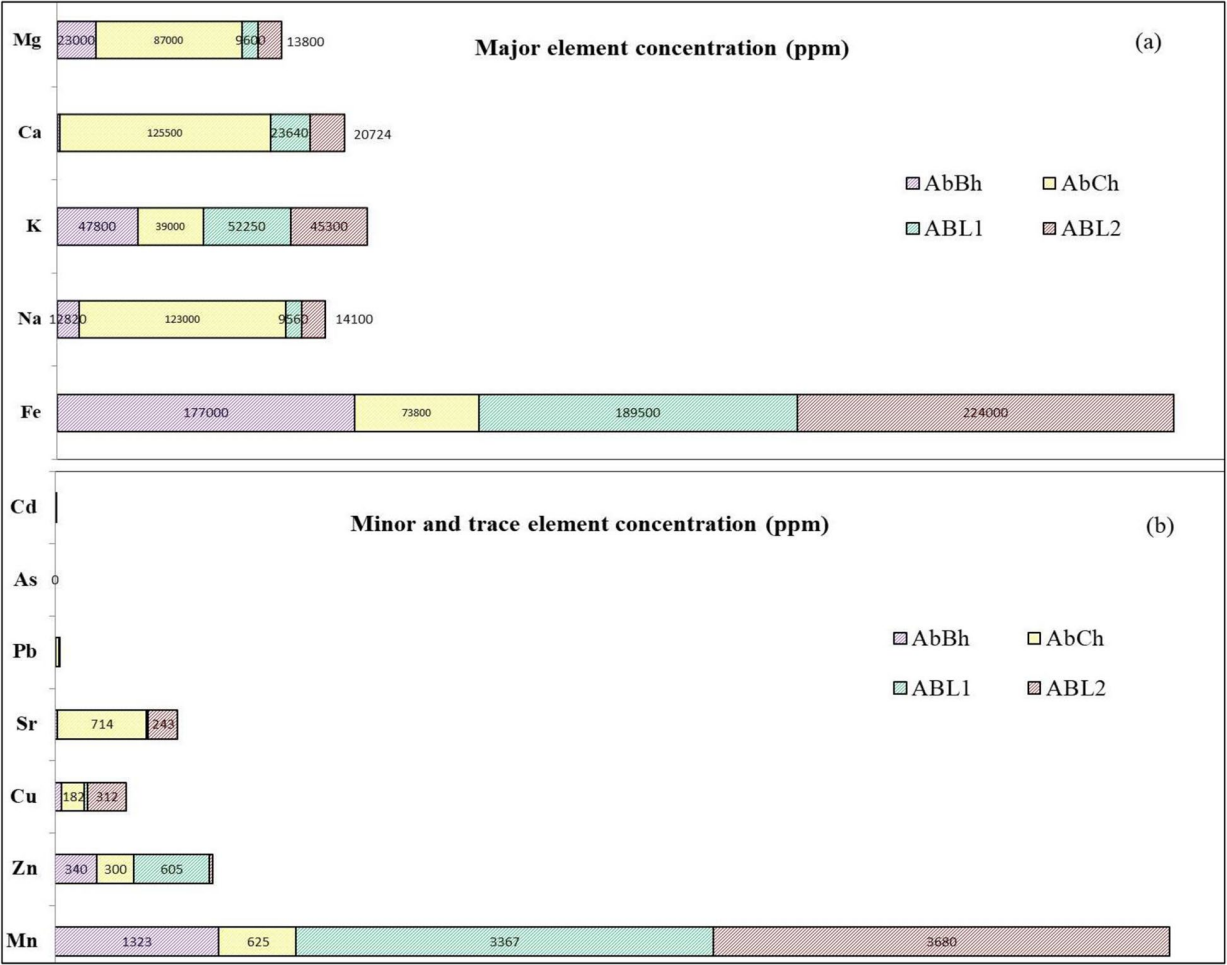
Compositions of (**a**) major elements (**b**) trace elements of ABL1, ABL2, AbBh and AbCh drugs

Among the cations measured by AAS, the nutritional elements such as Na, K, Ca and Mg, required for microbial functions [33], were detected in all four drug samples (Fig. 3). However, these major ions were present in considerable amounts in AbCh that was inactive against microbes. All the active drugs showed significantly high Fe concentrations, while the inactive drug AbCh had low Fe concentration. The metals Zn, Mn, Cu, Sr and Pb were identified as trace metals in the drug samples. The concentrations of Mn and Zn were relatively high in ABL1. Relatively high amounts of Cu and Pb were present in the inactive drug AbCh. The CEC values of ABL1, ABL2, AbBh and AbCh were 8.6, 8.4, 9.4 and 45.6 meq/100 g, respectively, which can be arranged in decreasing order as follows: CEC_AbCh_ > CEC_AbBh_ > CEC_ABL1_ > CEC_ABL2_.

### Analysis of interactions between bacteria and drugs by SEM

**Fig. 4:**
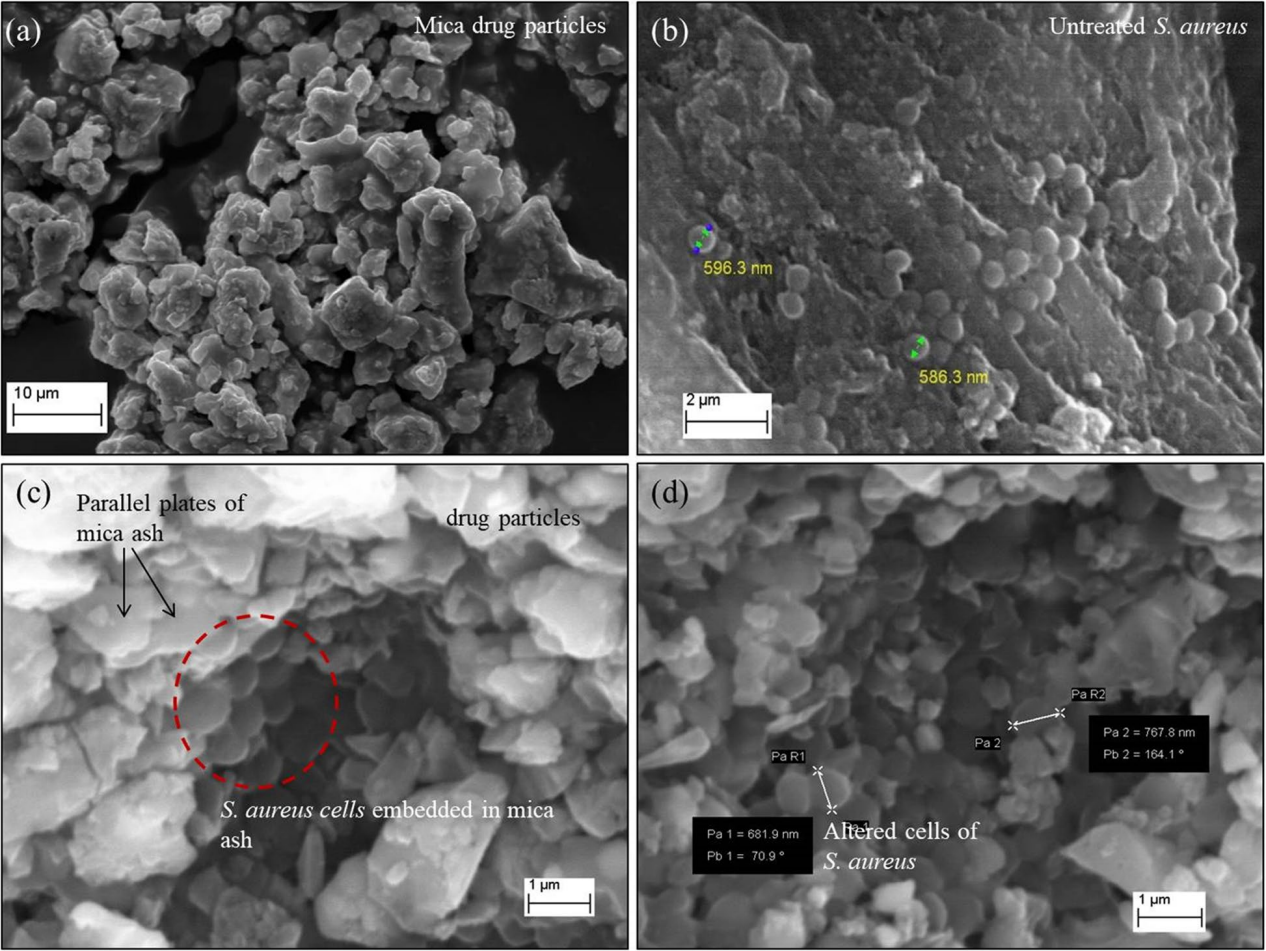
SEM images of (**a**) ABL1 drug rich in iron oxides and remnants of mica ashes (**b**) sphere-shaped cells of untreated *S. aureus* (**c**) cells of *S. aureus* embedded in parallel plates of mica ash (**d**) altered *S. aureus* cells adhered to the surface of ABL1 drug particles

The interactions between bacteria and drug particles were observed by SEM. The electron micrographs of bacteria (*S. aureus*) treated with ABL1 are shown in Fig. 4. spherical -shaped cells (cocci) of untreated *S. aureus* were observed in the blank sample (Fig. 4b). However, aggregates of morphologically altered *S. aureus* (circled area in Fig. 4c) were embedded in mica ash. Further, some of the microbes were adhered to the surface of the mineral particles of ABL1 (Fig. 4d). Altered microbes were relatively flattened and elongated in shape (Fig. 4d) compared to the spherical -shape observed in the untreated *S. aureus* (Fig. 4b).

### Cytotoxicity bioassay

None of the drugs caused time- or dose-dependent lethality to larvae in the brine shrimp cytotoxicity assay, indicating that the lethal dose of the water-soluble portion of the drugs is higher than that in the maximum amount of the drugs tested (10 mg/ml).

## Discussion

### Physical and chemical factors influencing antimicrobial activity of the drugs

SEM images reveal that *S. aureus* adhered to the surface of the active drug samples. Surface nature (surface roughness and charge) of the drug particles can influence the adhesion of the bacterial species to the surface of the drug particles [34]. Ion oxides are characterized by rough surfaces despite the smooth cleavage surfaces in mica and surface of clay minerals [17, 34, 35]. Therefore, the contact between mineral particles and the bacteria could be expected to be strong in the drugs that were rich in iron oxides (ABL1, ABL2 and AbBh) compared to AbCh that contained a relatively low amount of iron oxide (Fig. 3).

Electrostatic attraction between the microbes and the drug particles is another factor that would contribute to microbe lethality. Bacterial cell wall has a net negative charge both in Gram-positive and -negative bacteria due to the presence of teichoic acids (rich in phosphate), phospholipids and lipo-polysaccharides in their structure [33, 36]. Iron oxides on the other hand have a net positive charge over a wide range of pH [2–7] conditions. Negatively charged bacteria could readily adhere to the drug particles (ABL1, ABL2 and AbBh) that are rich in positively charged iron oxide sites [37, 38]. Drug particles of AbCh could be having a relatively low negative surface charge compared to the other drugs due to the presence of high fractions of clay minerals [39, 40]. The electrostatic repulsions between the microorganism and AbCh drug particles could be a factor contributing to the inactivity of AbCh drug.

Although electrostatic attractions could be expected in both Gram-positive and -negative bacterial species, the antimicrobial effect was observed in the current study only in Gram-positive bacteria. This difference in observation could be explained by the dissimilarities of Gram-positive and Gram-negative bacterial cell walls. The cell wall of Gram-positive bacteria has a thick layer of peptidoglycan while Gram-negative bacteria have a thick lipid bilayer on the outside, which is selectively permeable, in addition to the thin peptidoglycan layer. The peptidoglycan layers of Gram-positive bacteria are more permeable than the lipid bilayer of the Gram-negative bacteria. Due to this reason, Gram-positive bacteria are much more susceptible to antibiotics than Gram-negative bacteria [41–44]. Similarly, the antimicrobial cations (see below) of the drugs used in the current study are less likely to permeate through the lipid bilayer of Gram-negative bacteria than through the peptidoglycan layer of Gram-positive bacteria. Thus, the drugs were more effective against the Gram-positive organisms than the Gram-negative organisms. Further, drug permeability through the fungal membranes could be slower than the bacterial membranes due to the presence of the chitin cell wall in fungi, and this could be a reason for the drugs to be ineffective against *C. albicans.*

Although the physical properties of the drugs appear to influence antimicrobial activity, all the drugs were inactive when the tests were done using the solid growth media (well diffusion assay and agar dilution assay) probably due to the limited diffusion of material at a 10 mg/ml concentration. Chavadi [45] has observed antimicrobial activity of a mica drug at a much higher concentration (20 mg/ml) against *S. aureus* and *E. coli* by the well diffusion assay. Since the results of a diffusion assay could depend on the concentration and water-solubility of a particular antibiotic in addition to physical properties of the antibiotic, Chavadi’s results [45] observed in the well diffusion assay could be attributed to both physical properties and chemical constituents of the drugs. According to the results obtained in the current study where the broth dilution method (Miles and Misra) displayed antimicrobial activity (at 10 mg/ml), it could be that the drug particles were easily dispersed in water (broth) enabling better contact with the microbes when compared to the solid medium in the well diffusion assay.

When considering the antimicrobial results in relation to chemical constituents of the drugs, iron-rich drugs exhibited relatively high antimicrobial activity. Iron is present mostly as iron oxides in the drugs. However, the conventional iron oxide is not considered as an antimicrobial agent unless it is in the form of nanoparticles [36, 46]. In addition, iron oxides are known to be antibacterial when the metal ions are released to the bacterial environment in aqueous medium [1, 47]. The current results support the above contentions as the drugs showed their activity only when allowed to disperse in the aqueous BHI growth medium. This could be due to the greater interaction of drug particles with the microbes in liquid media. Further, antimicrobial cations released to the growth medium could easily penetrate through the bacterial cell wall in aqueous medium.

The CEC of a mineral indicates the amount of its exchangeable cations. If an antimicrobial mineral (specifically clay) carries effective antimicrobial cations, the mineral could act as a suitable antimicrobial agent; Fe^2+^, Zn^2+^, Mn^2+^, Cu^2+^ and Pb^2+^ cations have shown antimicrobial properties in previous studies [33, 48]. AbCh drug possess high CEC presumably due to the presence of significantly high clay fractions. However, its inactivity as well as growth enhancement in microorganisms indicate that the presence of relatively high amounts of exchangeable cations (Na^+^, K^+^, and Ca^2+^) in AbCh sustains and promotes the growth of rather than causing lethality to the microorganisms; Na^+^ ions are essential to the microorganisms for optimum growth [49] while Ca^2+^ and K^+^ ions can enhance the growth of microorganisms [50]. Hence, the antimicrobial activity of the investigated drugs of the current study is determined by their physical and chemical properties.

The doses of the drugs that are lethal to brine shrimp nauplii appear to exceed the 10 mg/ml level. Therefore, the water soluble preparations of the drugs at a concentration of 10 mg/ml could be used as promising antimicrobial agents devoid of cytotoxicity.

### Effect of the preparation technique of the drug on antimicrobial activity

Since the major mineral ingredient (biotite mica) used in the drug preparation was common to the four drugs, the differences in antimicrobial activity of the drugs could be due to the differences in the other ingredients used, and/or the method of drug preparation. The CEC, cationic constituents and mineralogical composition of the drug, which are important parameters for antimicrobial activity, depend on the preparation technique. Modern technology can be used to improve the traditional drug processing techniques to enhance the antimicrobial properties of mica drugs. This can be done in many ways, viz by increasing solubility and reducing particle size (nanometer range) of mica ash. Further, the antimicrobial properties of drugs with high CEC can be enhanced by the addition of cations such as Ag^+^, Cu^2+^, Zn^2+^ and Fe^2+^ which are known to have a high antimicrobial potential [16, 51].

## Conclusions and recommendations

Three mica-based drugs (*Abhrak Bhasma*, two prepared in the laboratory and one procured commercially) exhibited antimicrobial activity against *S. aureus* and MRSA (Gram-positive organisms) in aqueous medium, indicating the potential of these drugs to be developed as antimicrobial agents. The drugs did not show antibiotic activity against Gram-negative bacteria investigated nor antifungal activity against *C. albicans.* All four drugs were similar in terms of mineralogical composition. Chemical variables due to the method of drug preparation were significant, either to inhibit or promote microbial growth. Antimicrobial activity vs. cation exchange capacity showed an inverse relationship due to the ease of releasing cations from the drugs, which enhances microbial growth. Surface morphology and the preserved original platy nature of biotite in the drug particles can influence the adhesion and trapping of the bacterial species. Therefore, it could be stated that products based on mica ash exerted their lethal activity on bacteria by modifying the physiochemical characteristics directly through surface interactions or indirectly by changing the chemical properties of the growth medium. Antimicrobial parameters of the drugs such as particle size, aqueous solubility of antimicrobial cations and inducibility of morphological perturbations in microbial organisms should be further investigated to expound the inhibitory mechanisms and to grasp the full potential of mica-based drugs in clinical applications. Consistent antibacterial efficacy of the drugs should be ensured by standardization of the composition of raw materials and the techniques. Based on the results, it can be concluded that, antimicrobial activity of mica drugs could differ with the constituents of each drug and the method of preparation.

## Data Availability

The datasets used and/or analyzed during the current study are available from the corresponding author on reasonable request.

## Abbreviations

AbBh: *Abhraka Bhasma*
AbCh: *Abhrak Chenhuram*
ABL1: *Abhrak Bhasma* Laboratory Prepared 1
ABL2: *Abhrak Bhasma* Laboratory Prepared 2
ATCC: American Type Culture Collection
MRSA: Methicillin-resistant *Staphylococcus aureus*
DMSO: Dimethyl sulfoxide
MHA: Mueller-Hinton agar
BHI: Brain Heart Infusion
cfu: Colony forming units
XRD: X-ray diffraction
AAS: Atomic Absorption Spectrophotometer
CEC: Cation-exchange capacity
SEM: Scanning electron microscopy
